# TRIM14 inhibits hepatitis C virus infection by SPRY domain-dependent targeted degradation of the viral NS5A protein

**DOI:** 10.1038/srep32336

**Published:** 2016-08-31

**Authors:** Shanshan Wang, Yongzhi Chen, Chunfeng Li, Yaoxing Wu, Lei Guo, Changwei Peng, Yueping Huang, Genhong Cheng, F. Xiao-Feng Qin

**Affiliations:** 1 The Key Laboratory of Gene Engineering of the Ministry of Education and State Key Laboratory for Biocontrol, School of Life Sciences, Sun Yat-Sen University, Guangzhou, 510275, China; 2 Center for Systems Medicine, Institute of Basic Medical Sciences, Chinese Academy of Medical Sciences & Peking Union Medical College, Beijing 100005; Suzhou Institute of Systems Medicine, Suzhou, Jiangsu 215123, China; 3 CAS Key Laboratory of Infection and Immunity, Institute of Biophysics, Chinese Academy of Sciences, Chaoyang District, Beijing, China; 4 University of Chinese Academy of Sciences, Beijing, China; 5 Department of Microbiology, Immunology and Molecular Genetics, University of California, Los Angeles, CA 90095, USA

## Abstract

Tripartite motif 14 (TRIM14) was reported to function as a mitochondrial signaling adaptor in mediating innate immune responses. However, the involvement of TRIM14 in host defense against viral infection and molecular mechanisms remain unclear. Here, we demonstrated that enforced expression of TRIM14 could potently inhibit the infection and replication of HCV in hepatocytes, whereas TRIM14 knockout cells became more susceptible to HCV infection. Interestingly, further experiments revealed that such anti-HCV activity was independent of activating the NF-κB or interferon pathways but required the C-terminal SPRY domain of no signaling capacity. In searching for mechanisms how TRIM14 exerts its antiviral function we found that TRIM14 interacted with HCV encoded non-structural protein NS5A and could strongly induce its degradation dependent on the NS5A1 subdomain. Interestingly extensive domain mapping analyses revealed that NS5A degradation was mediated by the highly conserved SPRY domain of TRIM14, which might involve the K48 ubiquitination pathway. Collectively, our work uncovered a new mechanism responsible for host defense against HCV infection, and could potentially aid the development of novel anti-HCV therapeutics.

Hepatitis C virus (HCV), a single-stranded RNA, belongs to the Flaviviridae family, is an enveloped virus with a 9.6-kb genome^1^. The N-terminal segment of the polyprotein encodes structural proteins, consist of core protein and two glycoproteins E1 and E2, the C-terminal portion of the polyprotein contains nonstructural proteins p7, NS2, NS3, NS4A, NS4B, NS5A and NS5B^2^. HCV is one of major reasons that causes chronic liver disease including cirrhosis, steatosis and hepatocellular carcinoma^3^. Estimates show that about 180 million people are infected worldwide by HCV^4^,^5^,^6^. The standard of care for HCV infected patients was a combination of injected peg-related interferon alpha (peg-IFNα) and oral ribavirin administered for 48 weeks. HCV has some special characteristics, such as strong pathogenicity, no HCV preventive vaccine, poorly tolerated, frequently develop to liver cirrhosis and hepatocellular carcinoma (HCC)^7^. It is urgently needed to develop new strategies to combat with HCV.

NS5A is a HCV nonstructural protein, contains 448 amino acid (aa), composed of three domains (D1–D3) separated by two linker regions^8^. D1 is mainly attached to the inner-surface of phospholipid membrane^9^. D1 dimer includes a putative RNA-binding domain located at interface of the dimer^10^ and it forms a protective replication compartment that anchors the HCV RNA on intracellular membranes^11^. D2 is involved in binding to cyclophilin A and HCV RNA. D2 also can promote NS5A dimerization and it has the potential to play off against the innate immune response caused by HCV infection^12^,^13^. A recent study has demonstrated that D2 is required to suppress the activation of the interferon response^14^,^15^. D3 plays an important role in the assembly of infectious viral particles^12^,^13^.

The innate antiviral response represents the first line of host defense against viral infection^16^,^17^. When the host detected viral infection, cells can trigger a series of signaling events that lead to production of inflammatory cytokines and type I interferons (IFNs), such as IFN-α and IFN-β^18^,^19^. IFNs can induce the expression of ISGs and the ISGs play a central role in restricting virus replication^20^,^21^. The tripartite motif containing (TRIM) proteins have been implicated in many biological processes including cell differentiation, apoptosis, and transcriptional regulation^22^. Numbers of the tripartite motif (TRIM) proteins are increasingly recognized as ISGs which mediate antiviral responses^23^,^24^. Previous studies found that TRIM5α restricts human immunodeficiency virus (HIV-1) infection by TRIM5α PRYSPRY domains interaction with HIV-1 capsid core^25^. TRIM6 can interact with hepatitis B virus (HBV) core promoter to inhibit HBV RNA transcription^26^. TRIM11 can not only inhibit HIV-1 particle release but also inhibit murine leukemia virus (MLV) transcription^27^. TRIM19, as a mediator in IFN-α pathway, can inhibit replication of many kinds of virus, including herpes simplex virus (HSV-1), ebola virus (EBOV), lymphocytic choriomeningitis virus (LCMV), lassa virus (LASV), influenza A virus (IAV), vesicular stomatitis virus (VSV), rabies virus (RABV) and HIV-1^28^,^29^,^30^. TRIM22 has been shown to inhibit HIV-1 transcription^31^,^32^ or late events of the HIV-1 life cycle^33^. TRIM22 restricts a spectrum of DNA and RNA viruses, such as IAV, HCV^34^,^35^, encephalomyocarditis virus (EMCV) and HBV^36^,^37^.

Recently, TRIM14 was found as a mitochondrial adaptor mediated innate immune response by interacting with MAVS and NEMO^38^. TRIM14 contains a B-box, a coiled-coil, and a C-terminal B30.2/SPRY (PRYSPRY) domain but lacks the N-terminal RING domain. The 365^th^ amino acid site of TRIM14 is essential for the interaction between TRIM14 and NEMO, and the K365R mutant of TRIM14 could not up-regulate the NF-κB and type I interferon signaling pathway^38^. In that report, the authors found that TRIM14 could inhibit VSV virus replication by enhancing the type I interferon pathway and NF-κB production. However, the function of TRIM14 on HCV infection and replication in liver cells and the mechanism underline that are currently unknown. In the present study, we verified the inhibitory function of TRIM14 to HCV replication and infection on JFH cells and Huh7 cells. Interestingly, we found that the inhibitory function of TRIM14 to HCV replication is independent of its activity of up-regulate the type I interferon pathway and NF-κB production. Then, we established a Bi-molecular luminescence complementation (BiLC) assay to see whether TRIM14 could bind and target to any HCV proteins, and we found that TRIM14 mainly interact with NS5A and NS5B. Most interestingly, we demonstrated that TRIM14 interacted with NS5A and induced NS5A degradation in a dose dependent manner and was mainly mediated by SPRY domain.

## Results

### TRIM14 is induced by IFNs and can potently inhibit HCV replication

**Figure 1:**
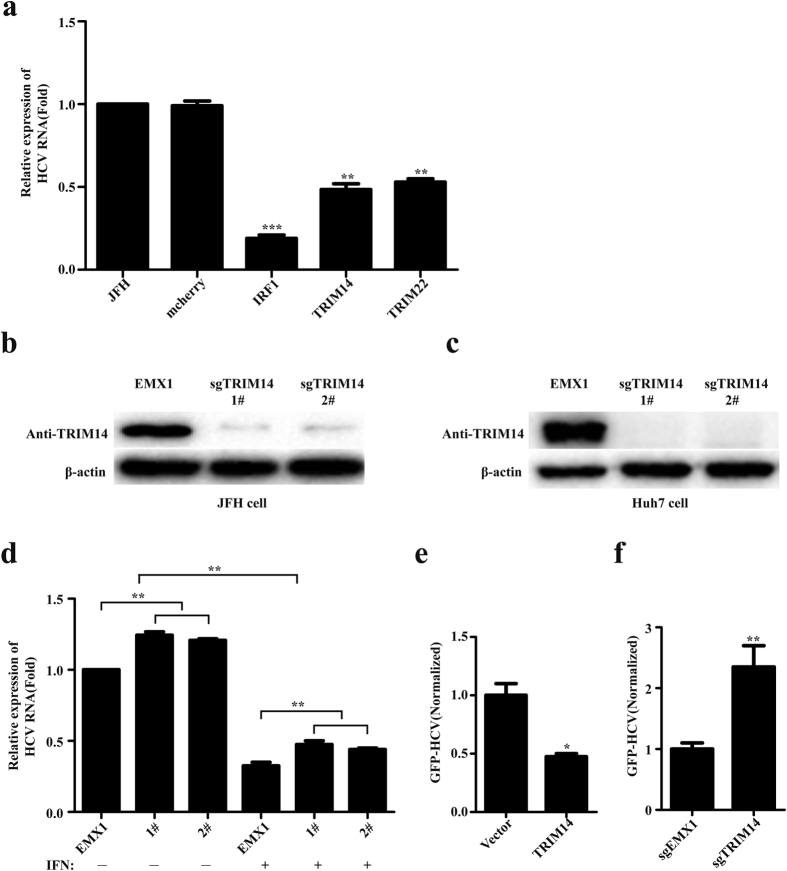
TRIM14 suppresses HCV infection and replication. (**a**) Effect of TRIM14 on HCV replication. TRIM14 was over expressed in JFH cells, quantitative real-time PCR method was used to detect the replication of HCV, mcherry served as a negative control, while IRF1 and TRIM22 as positive controls. (**b**) Immunoblot analysis of protein expression of TRIM14 in JFH knockout cells constructed by using CRISPR/Cas9. JFH monoclonal cells (1# or 2#) were harvested and analyzed by western blot to determine the knockout efficiency of TRIM14 in JFH cells. (**c**) Knockout of endogenous TRIM14 by CRISPR/Cas9 detected by western blot in Huh7 cells. (**d**) Effect of knockout of TRIM14 on HCV replication. Knockout of TRIM14 monoclonal cells (1# or 2#) were treated with or without IFN for 24 hours and analyzed by quantitative real-time PCR to determine the relative expression of HCV replicon. (**e**) Effect of TRIM14 on HCV-GFP infection. HCV-GFP was quantified by the calculated value of the percentage% GFP-positive population andMFI of GFP signal. (**f**) Effect of knockout of TRIM14 on HCV-GFP infection. HCV-GFP was quantified by the calculated value of the percentage% GFP-positive population and MFI of GFP signal. All experiments were performed in triplicates and data shown are representative of three independent experiments with SEM indicated by error bars. *P < 0.05; **P < 0.01; ***P < 0.001.

We first studied the induction of TRIM14 in the present of IFNs (IFN-α, IFN-β, IFN-γ), we found that TRIM14 could be induced by IFNs in varying degrees in Huh7.5, JFH, HEK293T, A549, Jurkat and THP1 cells (see Fig. S1 in the supplemental material), TRIM14 gene main induced by type I IFNs (IFN-α, IFN-β) in a dose-dependent manner especially in JFH cells and Huh7.5 cells (see Fig. S1 in the supplemental material). Then we studied the antiviral function of TRIM14 on HCV, the plasmid expressing of TRIM14 was transfected into JFH cells, quantitative real-time PCR experiments were performed to detect the mRNA of HCV. The result showed that overexpression of TRIM14 could inhibit the replication of HCV significantly (Fig. 1a). To determine the roles of endogenous TRIM14, we performed knockdown experiments with four short interfering RNA (TRIM14-siRNA) constructs targeting different sites of TRIM14 mRNA. Immunoblot analysis indicated that 1# and 4# TRIM14-siRNA constructs could efficiently inhibit the expression of endogenous TRIM14 in JFH cells no matter with or without IFN (see Fig. S2–a in the supplemental material) and the expression of TRIM14 plasmids were transfected in HEK293T cells (see Fig. S1 in the supplemental material). Then two different TRIM14-siRNA constructs (1# and 4#) were transfected into the JFH cells, quantitative real-time PCR experiments showed that knockdown of TRIM14 enhances the replication of HCV (see Fig. S2–b in the supplemental material). Further, CRISPR/Cas9 system was used to knockout TRIM14, immunoblot analysis indicated that both TRIM14-sgRNA plasmids could efficiently inhibit the expression of endogenous TRIM14 in JFH cells and Huh7 cells (Fig. 1b,c). Knockout of TRIM14 promotes the replication of HCV as well as TRIM14 knockdown results (Fig. 1b,d). The plasmid expressing TRIM14 was transfected into Huh7 cells, then the cells were infected with HCV GFP reporter virus (HCV-GFP), HCV replication was analyzed and measured by FACS based on GFP signals. Consistently, overexpression of TRIM14 restricted the infection of HCV and knockout of TRIM14 enhanced the infection of HCV (Fig. 1e,f). These results suggest that TRIM14 inhibits the replication of HCV and restricts the HCV infection.

### TRIM14 inhibits HCV replication independent of interferon signaling and NF-κB pathway

**Figure 2:**
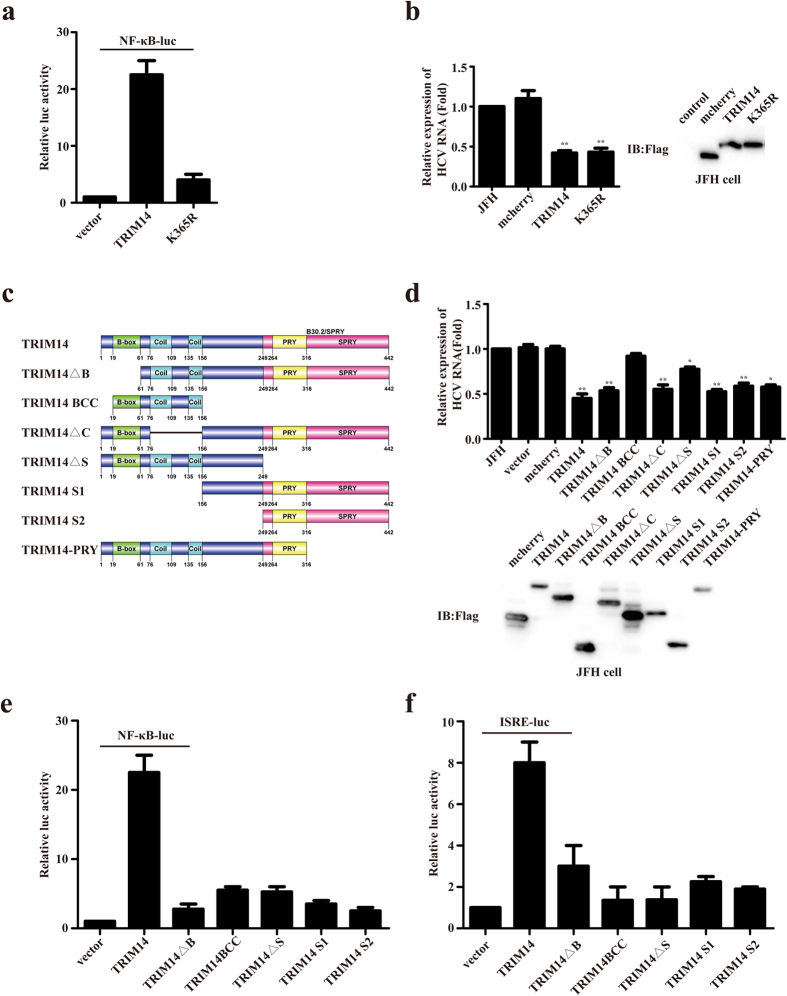
TRIM14 inhibits HCV replication not through NF-kB and ISRE pathway. (**a**) HEK293T cells were transfected with an NF-κB reporter plasmid as well as a control, TRIM14 or TRIM14 mutant K365R. (**b**) JFH cells were transfected with the expression plasmid for TRIM14 mutant (K365R), used quantitative real-time PCR method to detect the effect of K365R on the replication of HCV, and mcherry as a negative control. And immunoblot analysis of extracts of JFH cells which were transfected with expression plasmids for pFlag-TRIM14 and pFlag-K365R, mcherry as a control. (**c**) Schematic diagram of TRIM14 protein and TRIM14 truncations. (**d**) Effect of TRIM14 truncations on HCV replication. The TRIM14 truncations expression plasmids or a control plasmid were transfected into JFH replicon cells. After 48 hours, total RNA was analyzed for expression of HCV replicon and quantified by real-time PCR. Immunoblot analysis of extracts of JFH cells which were transfected with plasmids of pFlag-TRIM14 truncations (TRIM14ΔB, TRIM14BCC, TRIM14ΔC, TRIM14ΔS, TRIM14S1, TRIM14S2, TRIM14-PRY). (**e,f**) HEK293T cells were transfected with an NF-κB (**e**) or an ISRE reporter (**f**) plasmid as well as a control, TRIM14 or TRIM14 truncations (TRIM14ΔB, TRIM14BCC, TRIM14ΔS, TRIM14 S1, TRIM14S2) plasmids. Cells were harvested 24 hours later, and luciferase activity was analyzed.

Since an earlier report showed that TRIM14 could serve as a mitochondria adaptor to stimulate the IFN and NF-κB pathways^27^, we utilized K365R mutant form of TRIM to test whether the inhibitory function of TRIM14 to HCV replication is dependent on activation of IFN and NF-κB signaling. Consistent with the previously work, transfection of this mutant form of TRIM14 only induced a much lower level of NF-κB activation than wild type TRIM14 (Fig. 2a), nevertheless it showed equal potency in inhibiting HCV replication the wild type form of TRIM14 (Fig. 2b). These results indicated that TRIM14 could inhibit HCV replication by a novel mechanism, which is largely independent of NF-κB activation. Furthermore, in order to identify which domain of TRIM14 is responsible for its inhibition to HCV replication, TRIM14 truncations (TRIM14ΔB, TRIM14BCC, TRIM14ΔC, TRIM14ΔS, TRIM14S1, TRIM14S2, TRIM14-PRY) were constructed to test their inhibition of HCV replication (Fig. 2c). The plasmids expressing of TRIM14 truncations were transfected into JFH cells, and Red Fluorescent Protein (mcherry) as a negative control. Quantitative real-time PCR experiments results indicated that only TRIM14 truncations containing B30.2/SPRY domain could inhibit HCV replication, and the western blot showed that TRIM14 truncations were well expressed (Fig. 2d). To confirm that B30.2/SPRY domain could act on its own without the potential involvement of endogenous TRIM14 we performed the same experiment in TRIM14 knockout JFH cells. Results presented in Fig. S2–c (Supplemental Materials) showed that all TRIM14 truncations containing B30.2/SPRY domain could inhibit HCV replication with very similar potency in the knockout cells, thus B30.2/SPRY domain indeed can operate independent of the wild type TRIM14 protein in inhibiting HCV replication. To further support this, we found TRIM14 truncations (TRIM14ΔB, TRIM14BCC, TRIM14ΔS, TRIM14S1, TRIM14S2) had much lower activity on stimulating NF-κB as well as ISRE promoter compared with wild typeTRIM14 (Fig. 2e,f). Taken together, these results suggest that TRIM14 SPRY domain is essential for its inhibitory activity and it inhibits HCV replication by a novel mechanism, which is independent of up-regulating NF-κB or IFN signal pathways.

### TRIM14 interacts with NS5A

**Figure 3:**
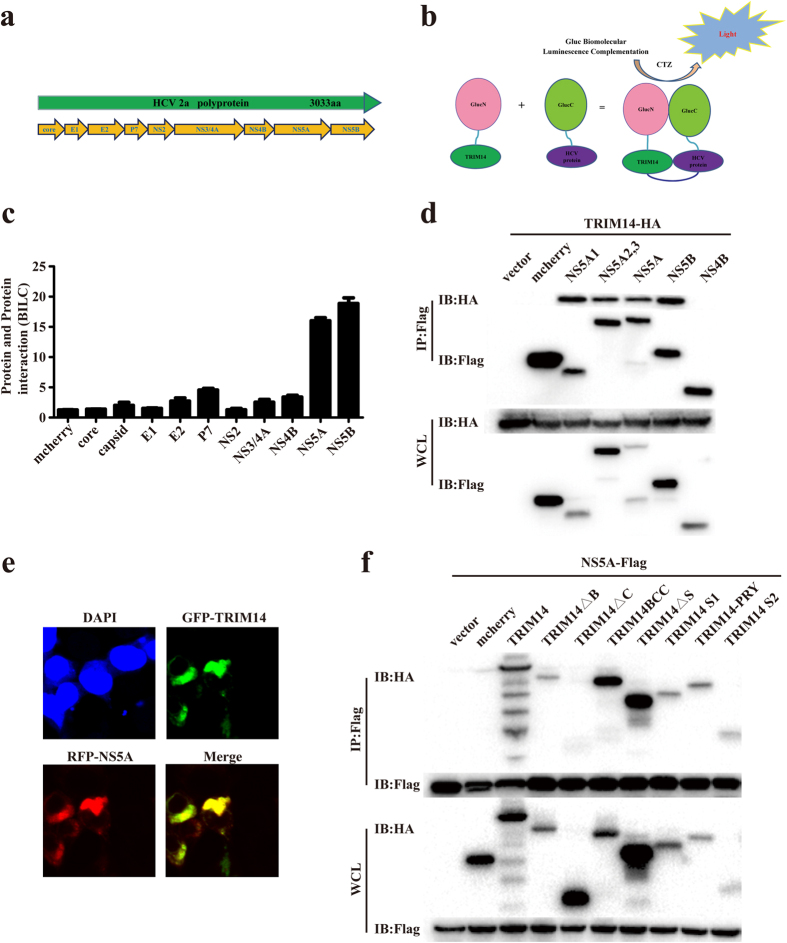
TRIM14 interacts with HCV NS5A. (**a**) HCV encoding proteins (core, E1, E2, p7, NS2, NS3/4A, NS4B, NS5A, NS5B). (**b**) Using BiLC system model to test the interactions between TRIM14 and HCV proteins. (**c**) Interactions between TRIM14 and HCV coding proteins were screened by a BiLC-based method. Each dot plot represents relative luminescence units from HEK293T cells over expression of individual pair of GlucN-HCV and GlucC-TRIM14 in HEK293T cells compared to controls, detected with Microplate System at 24 hours post transfection. (**d**) HEK293T cells were transfected with HA-tagged TRIM14 and Flag-tagged NS5A1, NS5A2.3, NS5A, NS5B, NS4B plasmids, mcherry as a negative control, 24 hours later, cell lysates were immunoprecipitated using anti-Flag, followed by western blot analysis using indicated antibodies. (**e**) Colocalization of TRIM14 with NS5A. Huh7 cells were co-transfected with GFP-TRIM14 and RFP-NS5A expression plasmids for 24 hours, then cell nucleus were stained with DAPI, then cells were detected by laser confocal microscopy. Red fluorescence shows NS5A staining, while green fluorescence detects TRIM14. (**f**) TRIM14 and TRIM14 truncations interacts with NS5A. Expression plasmids of pHA-TRIM14 or TRIM14 truncations were co-transfected with pFlag-NS5A constructs, mcherry as a negative control. Co-IP assays were performed using cell lysates from cells expressing the indicated constructs.

In order to investigate whether TRIM14 could bind to any HCV proteins to inhibit HCV replication, BiLC system based protein complementation assay was used to investigate TRIM14–HCV protein interactions. The *Gaussia* protein was splited into two parts GluN (17–109) and GluC (110–185), which mediated protein-protein interactions. TRIM14 were fused with GluN onto lentiviral vector using gateway recombination system. HCV proteins (Core, E1, E2, P7, NS2, NS3/4A, NS4B, NS5A and NS5B) were connected to GluC to produce GluC-HCV fusion proteins (Fig. 3a,b). In our BiLC screening study, TRIM14 specifically interacted with NS5A and NS5B which was further validated by Coimmunoprecipitation (Co-IP) experiments (Fig. 3c,d). In confocal microscopy experiments, TRIM14 and NS5A protein are more than 80% localization in the cytosol of Huh7 cells (Fig. 3e), the co- localization of TRIM14 and NS5A were more clear in a single cell (see Fig. S2–d in the supplemental material). Next we co-transfected the plasmids expressing TRIM14 truncations and NS5A into HEK293T cells, Co-IP results showed that B30.2/SPRY domain of TRIM14 was responsible for the interaction between TRIM14 and NS5A (Fig. 3f), and we co-transfected the plasmids expressing TRIM14 truncations and NS5A domain1 (NS5A1) into HEK293T cells, Co-IP results (see Fig. S3 in the supplemental material) showed in accordance with NS5A (Fig. 3f). Collectively, these results suggest that TRIM14 interacts with HCV protein NS5A, NS5B and TRIM14 truncations interact with HCV protein NS5A by NS5A1.

### TRIM14 can induce the degradation of NS5A

**Figure 4:**
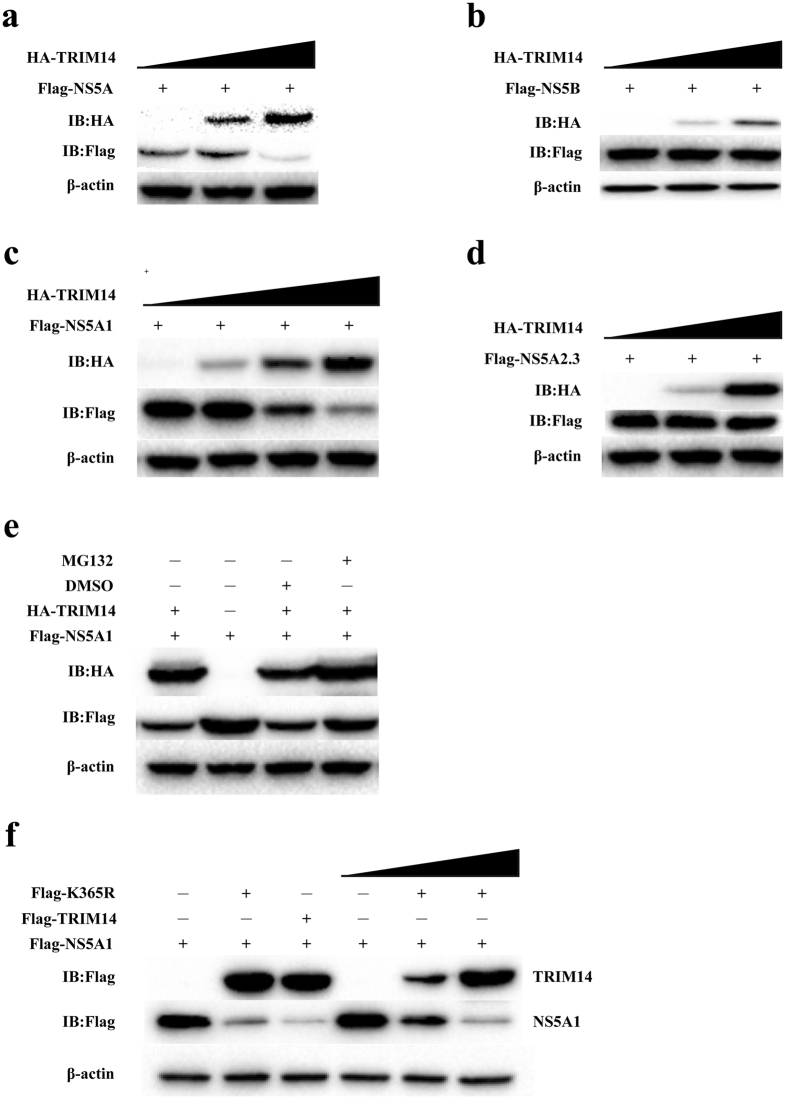
TRIM14 could induce the degradation of NS5A but not NS5B. (**a**) Effect of TRIM14 on degradation of NS5A. HEK293T cells were transfected with the pFlag-NS5A plasmids and an increasing amount of pHA-TRIM14 plasmids (0 ng, 300 ng or 600 ng, wedges) for 24 hours, cells were collected and lysates were probed as indicated. (**b**) HEK293T cells were transfected with pFlag-NS5B plasmids and an increasing amount (0 ng, 300 ng or 600 ng, wedges) of expression plasmids for pFlag-TRIM14. After 24 hours, cells were collected and lysates were probed as indicated. (**c**) Immunoblot analysis of extracts of HEK293T cells transfected with plasmid expressing pFlag-NS5A1 and pFlag-TRIM14, and increasing amount of TRIM14 plasmids (0 ng, 200 ng, 400 ng or 600 ng, wedges). (**d**) Immunoblot analysis of extracts of HEK293T cells which were transfected with expression plasmids for pFlag-NS5A2,3 and pHA-TRIM14, and increasing amount (0 ng, 300 ng or 600 ng, wedges) of expression plasmids for TRIM14. (**e**) Immunoblot analysis of extracts in HEK293T cells which were transfected with expression plasmids for pFlag-NS5A1, pHA-TRIM14 or a control plasmid, then cells were treated with dimethyl sulfoxide (DMSO; vehicle) or MG132 for 6 hours. (**f**) Immunoblot analysis of extracts in HEK293T cells which were transfected with expression plasmids for pFlag-NS5A1and pFlag-K365R, pFlag-TRIM14 as a control, and increasing amount (0 ng, 200 ng, 400 ng or 600 ng, wedges) of expression plasmids for TRIM14 for 24 hours.

To study the effects of TRIM14 to NS5A and NS5B caused by the interaction between TRIM14 and NS5A or NS5B, we firstly investigated the stability of the HCV proteins in presence of TRIM14. TRIM14 and NS5A or NS5B were co-transfected into HEK293T cells. The western blot results demonstrated that TRIM14 could induce the degradation of NS5A in a dose-dependent manner while NS5B was not affected (Fig. 4a,b). To address whether TRIM14 might NS5A expression at the mRNA level, we performed quantitative real-time PCR experiment (Fig. S4 in the supplemental materials). The result showed that co-transfection of TRIM14 did not affect NS5A mRNA expression. therefore TRIM14 mediated NS5A degradation mainly occurred at the protein level.

We next examined the domain involvement of NS5A, the result showed that the degradation of NS5A induced by TRIM14 requires NS5A domain1 but not NS5A domain2 and 3 (NS5A2.3) (Fig. 4c,d). To further investigate whether TRIM14 mediated NS5A1 degradation was proteosome dependent, proteasome inhibitor MG132 was used to treat the cells co-expressing TRIM14 and NS5A1. The western blot results showed that the degradation of NS5A1 was rescued by MG132 (Fig. 4e). Since TRIM14 was reported to activate innate immune response through interacting with MAVS and NEMO and the K63-linked ubiquitination of TRIM14 at K365 plays an important role in the process^38^. So that in order to investigate whether the inhibitory function of K365 was as same as TRIM14 which was induced the degradation of NS5A1 in a dose dependent manner, we transfected HEK293T cells with pFlag-K365R, pFlag-NS5A1, pFlag-TRIM14 or empty vector, immunoblot results showed that overexpression of TRIM14 K365R mutants also can induce the degradation of NS5A1 in a dose dependent manner (Fig. 4g). It suggests that TRIM14 mediated NS5A degradation is dependent on proteosome pathway, but not through respond to NF-κB or ISRE signal pathway.

### B30.2/SPRY domain of TRIM14 plays an important role in inducing the degradation of NS5A1

**Figure 5:**
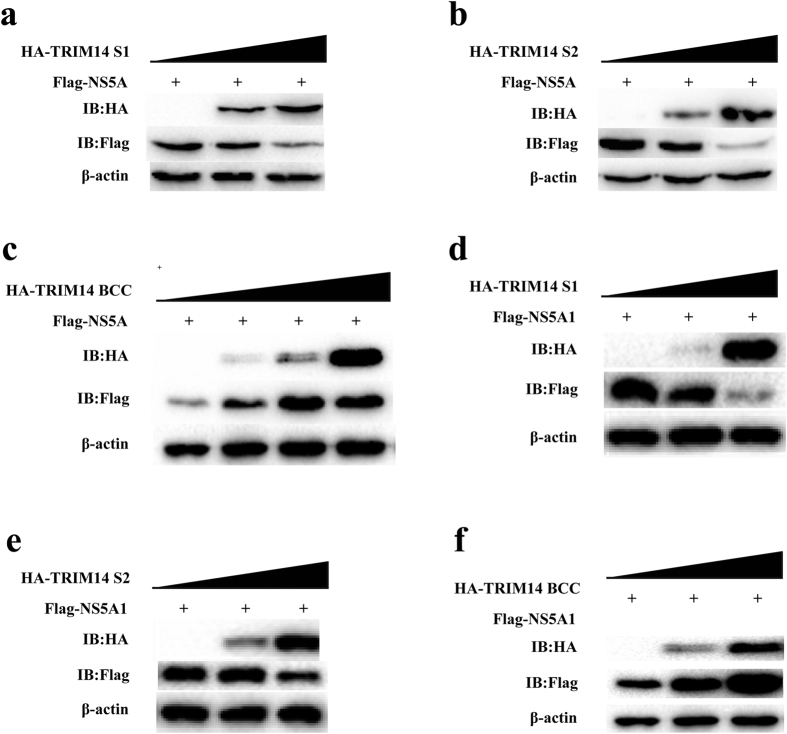
TRIM14 truncations interacts with HCV NS5A1. (**a–c**) Immunoblot analysis of extracts from HEK293T cells which were transfected with plasmids of pFlag-NS5A and pHA-TRIM14 truncations (TRIM14S1, TRIM14S2 or TRIM14 BCC), and increasing doses of these expression plasmids, such as TRIM14S1 and TRIM14S2, increasing doses of expression plasmids (0 ng, 300 ng or 600 ng, wedges) for 24 hours, and increasing amount (0 ng, 200 ng, 400 ng or 600 ng, wedges) of expression plasmids for TRIM14BCC. (**d,e**) Immunoblot analysis of extracts from HEK293T cells which were transfected with the plasmids of pFlag-NS5A1 and pHA-TRIM14 truncations (TRIM14S1, TRIM14S2 or TRIM14BCC), and increasing doses of expression plasmids (0 ng, 300 ng or 600 ng, wedges) for 24 hours.

The inhibitory effects of TRIM14 truncations (TRIM14ΔB, TRIM14ΔC, TRIM14ΔS, TRIM14BCC, TRIM14S1, TRIM14S2, TRIM14-PRY) were detected by quantitative real-time PCR experiments, and the results indicated that only TRIM14 truncations containing B30.2/SPRY domain can inhibit HCV replication (Fig. 2d). Next we co-transfected the plasmids expressing TRIM14 truncations and NS5A into HEK293T cells, Co-IP results showed that B30.2/SPRY domain of TRIM14 was responsible for the interaction between TRIM14 and NS5A (Fig. 3f). To test whether TRIM14 truncations (TRIM14BCC, TRIM14S1 and TRIM14S2) can degrade NS5A, we co-transfected the plasmids expressing TRIM14 truncations and NS5A into HEK293T cells, immunoblot results showed TRIM14 truncations TRIM14S1 and TRIM14S2 which contains B30.2/SPRY domain can induce the degradation of NS5A (Fig. 5a,b). On the contrary, TRIM14BCC without B30.2/SPRY domain promoted the expression of NS5A (Fig. 5c). It is the same that TRIM14S1 and TRIM14S2 can degrade NS5A1 but not TRIM14BCC (Fig. 5d–f). These results suggest that B30.2/SPRY domain is important for TRIM14-NS5A interaction and inhibitory function of TRIM14 to HCV replication.

### TRIM14 promotes ubiquitination of NS5A

**Figure 6:**
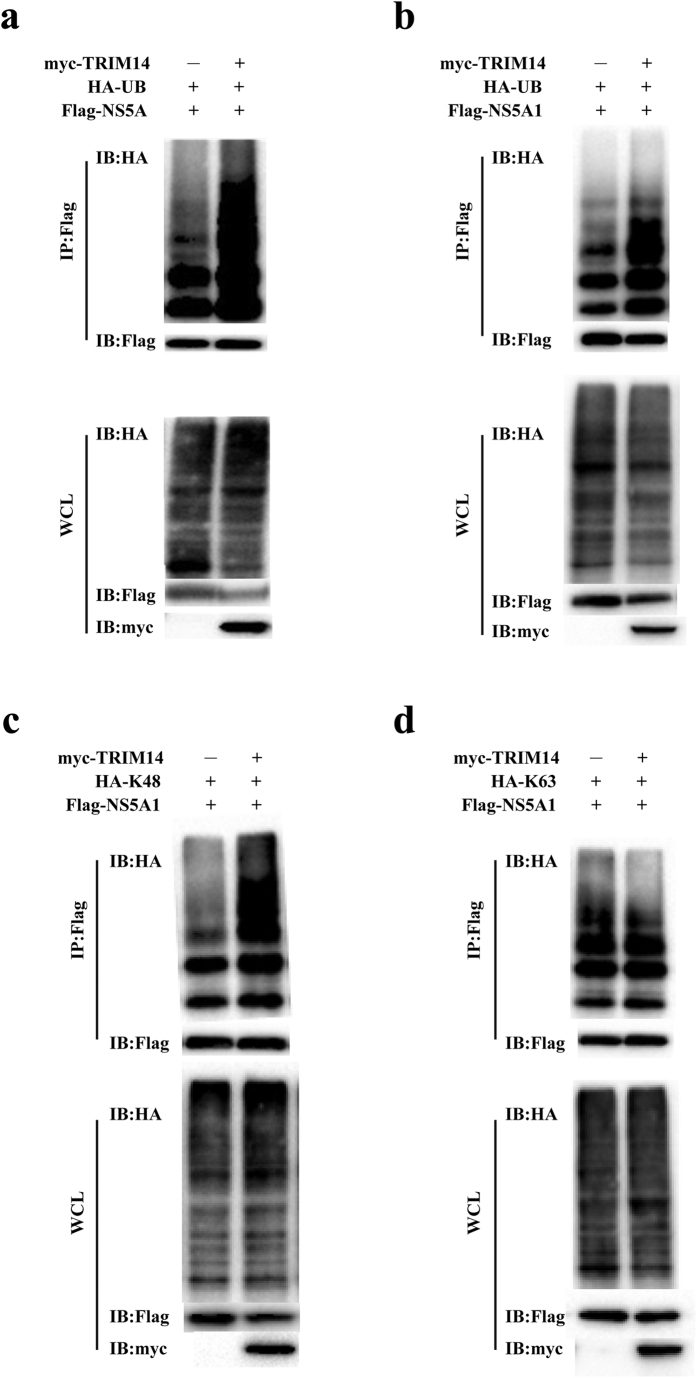
TRIM14 and its truncations induce NS5A1 by the way of the K48-linked ubiquitination. (**a,b**) Immunoassay of extracts from HEK293T cells which were transfected with various combinations of plasmids of pFlag-NS5A, pFlag-NS5A1, pHA-Ub, pMyc-vector or pMyc-TRIM14 followed by immunoprecipitation with anti-Flag beads and immunoblot analysis with anti-HA antibody. (**c,d**) Immunoassay of extracts from HEK293T cells which were transfected with various combinations of plasmids of pFlag-NS5A1, pMyc-TRIM14, pHA-K48-linked ubiquitination or pHA-K63-linked ubiquitination followed by immunoprecipitation with anti-Flag beads and immunoblot analysis with anti-HA antibody.

To examine whether TRIM14 mediated the degradation of NS5A might operate through ubiquitin modification, we performed standard co-transfection based ubiquitination assay. Thus pFlag-NS5A was co-transfected with pHA-Ub, pMyc-TRIM14 or empty vector, then coimmunoprecipitation and immunoblotting analyses were conducted, The result showed that overexpression of TRIM14 indeed could promote HCV NS5A protein ubiquitination (Fig. 6a). Similar experiment was performed with the NS5A1 subdomain construct next. The result showed that overexpression of TRIM14 could strongly promote NS5A1 ubiquitination as well (Fig. 6b). As it has been well established that K48-linked ubiquitination predominantly facilitated the proteasome mediated degradation of the protein substrates, whereas K63-linked ubiquitination mediated mainly cellular signaling functions, we therefore performed K48- and K63- specific ubiquitination assays. The results showed that overexpression of TRIM14 promoted K48-linked ubiquitination of NS5A1, but had little effects on the K63-linked ubiquitination of NS5A1 (Fig. 6c,d). Thus, together these studies demonstrate that NS5A degradation induced by TRIM14 correlates with the K48-linked ubiquitination.

## Discussion

The antiviral action of type I Interferon is the first defense line of the host through inducing ISGs to inhibit virus infection. ISGs work to restrict viral infection through many mechanisms, however, only a few ISGs have so far been defined to have anti-HCV properties. An understanding of the mechanisms by which these ISG effectors exert their inhibitory activities against HCV will not only help in the treatment modification to enhance the antiviral activity of IFNs-based therapy toward sustained virologic response, but also generat potential targets for novel antiviral therapy applications.

In this work, we demonstrated that TRIM14 functions as an interferon inducible gene that can potently inhibit HCV infection and replication (Fig. 1a,g). TRIM14 is one of the family members of the tripartite motif containing (TRIM) proteins that have been implicated in many biological processes including cell differentiation, apoptosis, and transcriptional regulation.

TRIM14 protein consists of B2, CC, B30.2/SPRY (PRYSPRY) domains, but does not contain RING domain, that can be found in other TRIM family members, which mediates activation of NF-κB signaling because of its E3 ubiquitin ligase activity^39^. B-box domains are important features of the TRIM proteins. B-boxes can have two different zinc binding motifs. Type I B-box (B-boxI) and Type 2 B-box (B-box2), and TRIM14 protein has Type 2 B-box domain. B-box2 may have a role in the regulation of the RING domain function or in tandem with the B-boxI domain, possibly by influencing substrate recognition and/or ubiquitin ligase activity. B-box2 may be important for the overall ability of proteins to form homo-multimers and interact with other proteins^23^,^40^,^41^. The coiled-coil motif of TRIM proteins is approximately 100 residues long and is frequently broken up in two or three separated coiled-coil sub-motifs. The coiled-coil domain is mainly involved in homo-interactions and in promoting the formation of high molecular weight complexes^40^. In this work, our that isolated BCC domain of TRIM14 appeared to increase the expression or stablilization of NS5A protein (Fig. 5c,f), we speculate that the BCC domain on its own might enhance the ability of NS5A proteins to form homo-multimers, thus more stable high molecular weight complexes. However, in the context of full-length protein of TRIM14, this activity might be restricted and subdued to the dominant ubiquitin modification function, nevertheless, it is possible that under certain conditions, BCC domain in full-length of TRIM14 can modulate the overall stability of NS5A.

Several recent large-scale functional screens have identified key ISGs with antiviral activity toward infectious HCV, complementing the previously identified a range of ISGs with activity against HCV subgenomic replicon^42^,^43^. While many ISGs have been identified with anti-HCV activity, the antiviral mechanisms of action toward HCV for many of these ISGs have yet to be elucidated. High expression levels of TRIM14 have also been reported in HIV-associated human and simian immunodefciency virus (SIV)-associated monkey lymphomas^44^. Enhanced expression of TRIM14 gene can suppress Sindbis virus reproduction by increasing the transcription of many genes involved in innate immunity^45^. In our experiment, we found that TRIM14 could be induced by interferon. Overexpression of TRIM14 could inhibit HCV infection and replication, while TRIM14 knockout cells were more susceptible to HCV infection and replication.

To further elucidate the molecular mechanisms of antiviral activity of TRIM14 on HCV, BiLC system was used to study the protein-protein interaction between TRIM14 and HCV encoded proteins, we found TRIM14 mainly interact with NS5A and NS5B, and TRIM14 can promote the degradation of NS5A not NS5B in dose dependent manner (Fig. 4a,b). TRIM14 was previously reported as a mitochondrial adaptor mediating innate immune response through interacting with MAVS and NEMO, and the activation of downstream NF-κB and IFN pathways. However, our studies showed that overexpression of K365R mutant of TRIM14 which had much lower activity in stimulating NF-κB signaling than TRIM14 wild type, had equal potency in inhibiting HCV replication and NS5A protein degradation (Fig. 4f) suggesting that signal activation function of TRIM14 is dispensable for HCV inhibition, while modulating NS5A protein level plays the key role.

It has been reported that the B30.2/SPRY domain of TRIM14 is critical for the interaction of TRIM14 with other proteins^38^, consistently, we also found that the truncations which has the B30.2/SPRY domain can inhibit the replication of HCV and interact with NS5A (Figs 2d and 3f). In addition, we showed that TRIM14 also interacted with NS5B, while NS5B could not be degraded. The biological effect of this interaction on HCV infection remains to be further investigated.

In summary, our findings indicate that TRIM14 could inhibit HCV replication through its interaction with NS5A, which leads to the degradation of NS5A in the K48-linked ubiquitination dependent way. Taken together, this work leads to better understanding the mechanisms responsible for host defense against HCV infection, also provides molecular basis for the potential development of novel anti-HCV therapeutics.

## Methods

### Reagents and Antibodies

Recombinant Human IFN-α, IFN-β, IFN-γ (Peprotech), Monoclonal anti-Flag M2-Peroxidase (Sigma), anti-HA-peroxidase (Roche), Mouse anti-beta actin antibody (Genscript), and rabbit anti-TRIM14 antibody was raised against recombinant human full-length TRIM14 (Aviva Systems Biology), MG132, G418, polybrene and dimethyl sulfoxide (DMSO) were purchased from Sigma.

### Plasmids and Expression Vectors

Mammalian expression plasmids for human pFlag-tagged, pHA-tagged, or pMyc-tagged TRIM14 were constructed by standard molecular biology techniques, TRIM14 truncations (TRIM14ΔB, TRIM14BCC, TRIM14ΔC, TRIM14ΔS, TRIM14 S1, TRIM14S2, TRIM14-PRY, K365R), were PCR and cloned into pL-EF1a-DEST-SFB-iP, pCMV-Flag, pCMV-HA to generate Flag/HA tagged expression constructs, TRIM14S1, TRIM14S2 were made from gene synthesis. HCV proteins (core, E1, E2, p7, NS2, NS3/4A, NS4B, NS5A, NS5B) were PCR and cloned into FG-EH-MCS/DEST-GlucC, FG-EH-GlucC-MCS/DEST, TRIM14 were cloned into FG-EH-GlucN-MCS/DEST, FG-EH-MCS/DEST-GlucN, NS4B, NS5A, NS5A1, NS5A2,3, NS5B were PCR and cloned into pCMV-Flag, pCMV-HA to generate Flag/HA tagged expression constructs.

### Cell culture and transfection

HEK293T, A549, Huh7, THP1, Jurkat cells were cultured in DMEM/RPMI medium 1640 (Hyclone) supplemented with 10% Fetal Bovine Serum (Gibco), 100 units/ml penicillin, 100 μg/ml streptomycin (Gibco) at 37 °C in an atmosphere of 5% CO_2_. JFH cells were maintained in complete DMEM supplemented with 500 mg/ml G418 (Sigma). Huh7 cells were maintained in complete DMEM supplemented with 1% non-essential amino acids. Expression vectors plasmid were transfected using Lipofectamine3000 (Invitrogen), Fugene6 (Promega) following the manufacturer’s protocol.

### Quantitative real-time PCR

The indicated ISG-expression plasmids were transfected into JFH replicon cells. After 48 hours, the first step, cells total RNA using Trizol reagent (Invitrogen) was extracted from JFH cells, the second step using Takara reverse transcription kits do experiment to obtain cDNA, then quantified by real-time PCR with Power SYBR Green PCR master mix (Roche) in an Roche fluorescence quantitative Real-time PCR system, the real-time PCR primer sequences were as follow: JFH:TCTGCGGAA CCGGTGAGTA (forward), TCAGGCAGTACCACAAGGC (reverse), GAPDH: GAACGGGAAGCTCACTGG (forward), GCCTGCTTCACCACCTTCT (reverse), TRIM14:TGAAGGGGAAATTCACTGAACTC(forward), AGCCTCTGGACAGGA TCGG (reverse). NAS5A:TATCAATTGCTACACGGAGGG (forward), CAGATTG TCAGTGGTCAGTCC(reverse).

### Knockdown and knockout TRIM14 gene

Design and synthesis of siRNA for knockdown TRIM14 protein expression:

1#: CAGAUUACUACUUGACGAA, 2#: GCUAAUGCAGAGUCAAGUA, 3#: UCCAGAGGCUUCAGGCAUA, 4#: CAACAUAACCCAGAUAGAA, four strip of siRNA were transfected to HEK293T cells, western blot select the best one for experiment. Design and construct sgRNA for knock out TRIM14 in genome: sgRNA1#: CACCGATCGTGTCAGGACAGCGT (forward), AAACACGCTGGATCCTGACAC GATC (reverse), sgRNA2#: CACCGCATCGTGTCAGGATCCAGCG (forward), AAACCGCTGGATCCTGACACGATGC (reverse). Cloned the sgRNA into pB-sgRNA vector, and CRISPR/Cas9 was cloned into FG-EH-DEST-2F-PPW vector. For each well of a 24-well plate, the cells were transfected with CRISPR/Cas9 (300 ng) and sgRNA (300 ng) expression plasmids by Lipofectamine3000. 48 hours later, cells were seeded into 96-well plates at the density of 0.5 cell/well with 100 μl DMEM. After amplification for 2–3 weeks, the TRIM14^*−/−*^ cells were verified by western blot.

### Flow cytometry

Effect of overexpression of individual ISGs on HCV-GFP in RFP-positive population normalized to vector control. Cells were collected by trypsinization, fixed in 2% paraformaldehyde and analyzed on BD FACS Calibur flow cytometer (Biosciences). HCV-GFP was quantified by the product of percent GFP-postive population and geometric mean of the fluorescence index^21^.

### Immunofluorescence and Co-immunoprecipitation

Huh7 cells were transfected with expression plasmids (500 ng) by lipofectamine 3000 (Invitrogen), after transfection 36 hours, the cells were washed with PBS, and fixed with 4% paraformaldehyde for 15 min at 4 °C. With methanol permeability at −20 °C, 10 min, nuclear was stained with DAPI for 10 min, then, slides were observed with a TCS-SP5 confocal microscope.

HEK293T cells (2 × 10^5^) were plated into 24-well plates and transfected with the expression plasmids (500 ng) by lipofectamine 3000, 24 hours later, the cells were washed with PBS, then cells were lysed in 250 μl of lysis buffer (20 mM Tris, pH 8.0, 150 mM NaCl, 1% TritonX-100 containing cocktail, 1 mM EDTA). Lysates were incubated with the appropriate sepharose beads (GE Healthcare) for 6–8 hours, in 360 degree rotating shaker, at 4 °C, sepharose beads were washed four times with 800 μl of lysis buffer, cell samples were running in SDS–PAGE and transferred to PVDF membranes according to standard immunoblot procedures.

### BiLC reporter assay

TRIM14 and HCV proteins-BiLC reporter constructs were cloned into the lentiviral vector backbone FG11F plasmid (Patent US20120201794 A1). GlucN (17-93aa) and GlucC (94-185aa) fragments (GenBank: AY015993) were inserted between the enzyme restriction sites AscI and RsrII of the FG11F vector. TRIM14 or HCV proteins were cloned into BiLC reporter system via the Gateway recombination cloning system (Invitrogen). HEK293T cells were seeded in 24-well plates the day before transfection. The lentiviral FG-EH-TRIM14-GlucN and FG-EH-HCV protein-GlucC plasmids were co-transfected into HEK293T cells, 48 hours later, the TRIM14 and HCV proteins-BiLC lentiviral particles were harvested, and luciferase activity was analyzed.

### Luciferase reporter assays

HEK293T (5 × 10^4^) cells were plated in 96-well plates and transfected with plasmids encoding an NF-κB (Firefly luciferase plasmid: 5 ng), and pRL-TK (Renilla luciferase plasmid: 2 ng) together with 100 ng plasmid encoding pFlag-TRIM14 and pFlag-TRIM14 truncations. Cells were harvested at 36 hours after transfection in passive lysis buffer (Promega). Cell lysates were measured using the dual luciferase assay kit according to the manufacturer’s protocol (Promega). The firefly luciferase activities were normalized to Renilla luciferase activities^22^,^46^.

### Statistical methods

GraphPad Prism 5 software (GraphPad Software, San Diego, CA) was used for data analysis using a two-tail unpaired *t* test. A p value < 0.05 was considered statistically significant.

## Additional Information

**How to cite this article**: Wang, S. *et al*. TRIM14 inhibits hepatitis C virus infection by SPRY domain-dependent targeted degradation of the viral NS5A protein. *Sci. Rep.* **6**, 32336; doi: 10.1038/srep32336 (2016).

## Supplementary Material

Supplementary Information
