# Supplementary material for: TRIM14 inhibits hepatitis C virus infection by SPRY domain-dependent targeted degradation of the viral NS5A protein
Source: Sci Rep. 2016 Aug 31;6:32336. doi: 10.1038/srep32336 (PMC5006124; doi:10.1038/srep32336)
Supplement: Supplementary Information [file srep32336-s1.pdf]

TRIM14 inhibits hepatitis C virus infection by SPRY domain-dependent targeted degradation of the viral NS5A protein

Shanshan Wang[a,b], Yongzhi Chen[c,d], Chunfeng Li[b], Yaoxing Wu[a], Lei Guo[a], Changwei Peng[a], Yueping Huang[a], Genhong Cheng[b,c,e], F. Xiao-Feng Qin[a,b]

The Key Laboratory of Gene Engineering of the Ministry of Education and State Key Laboratory for Biocontrol, School of Life Sciences, Sun Yat-Sen University, Guangzhou, 510275, China[a];

Center for Systems Medicine, Institute of Basic Medical Sciences, Chinese Academy of Medical Sciences & Peking Union Medical College, Beijing 100005; Suzhou Institute of Systems Medicine, Suzhou, Jiangsu 215123, China[b];

CAS Key Laboratory of Infection and Immunity, Institute of Biophysics, Chinese Academy of Sciences, Chaoyang District, Beijing, China[c];

University of Chinese Academy of Sciences, Beijing, China[d];

Department of Microbiology, Immunology and Molecular Genetics, University of California, Los Angeles, CA 90095, USA[e].

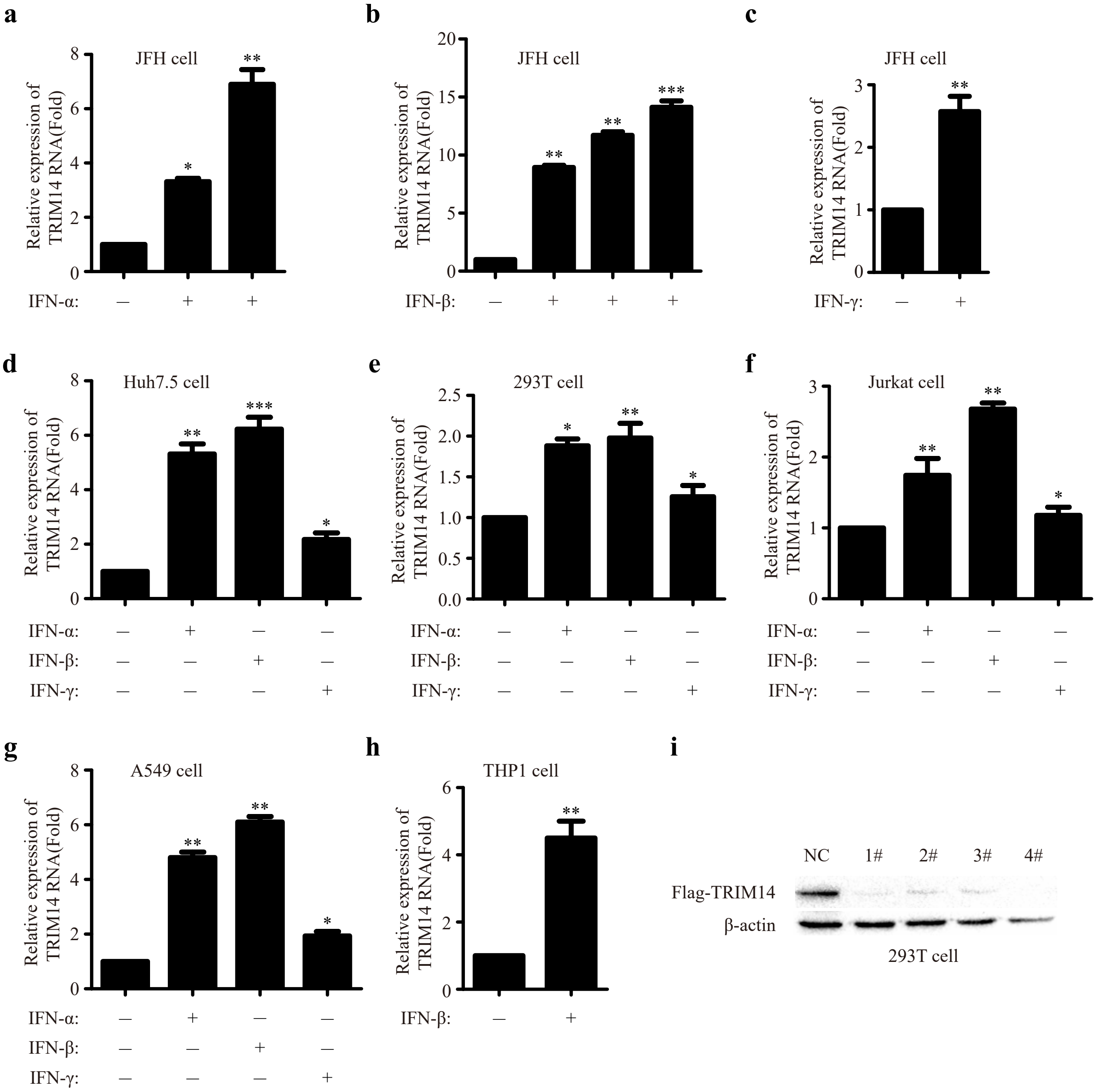

Supplemental Figure 1. TRIM14 is induced by IFN-α, IFN-β and IFN-γ

(a)-(c) The relative expression of TRIM14 was up regulation by IFNs (IFN-α, IFN-β, IFN-γ) in JFH cells. **(a)** Using different concentration of IFN-α induced in JFH cells, (IFN-α: 0IU, 10IU, 100IU, 1000IU). **(b)** Added different concentration of IFN-β (0ng, 10ng, 100ng) into JFH cells, and the expression of TRIM14 in a dose dependent manner. **(c)** Added IFN-γ (10ng) into JFH cells for 12 hours, total RNA

was analyzed using real-time quantitative PCR to detect the relative expression of TRIM14, the result indicated that TRIM14 can be induced by IFN-γ. **(d)-(h)** Quantitative assay detected the up regulation of TRIM14 induced by IFNs (IFN-α, IFN-β, IFN-γ) in Huh7.5 cells, HEK293T cells, Jurkat cells, A549 cells, THP1 cells. The relative expression of TRIM14 in different multiple change in cells. **(i)** First, we transfected pFlag-TRIM14 plasmid (300ng) in HEK293T cells, 12 hours later, HEK293T cells were transfected with siRNA oligos 1#, 2#, 3# and 4# for the second time, then immunoblot analysis the efficiency of knockdown of TRIM14 in HEK293T cells.

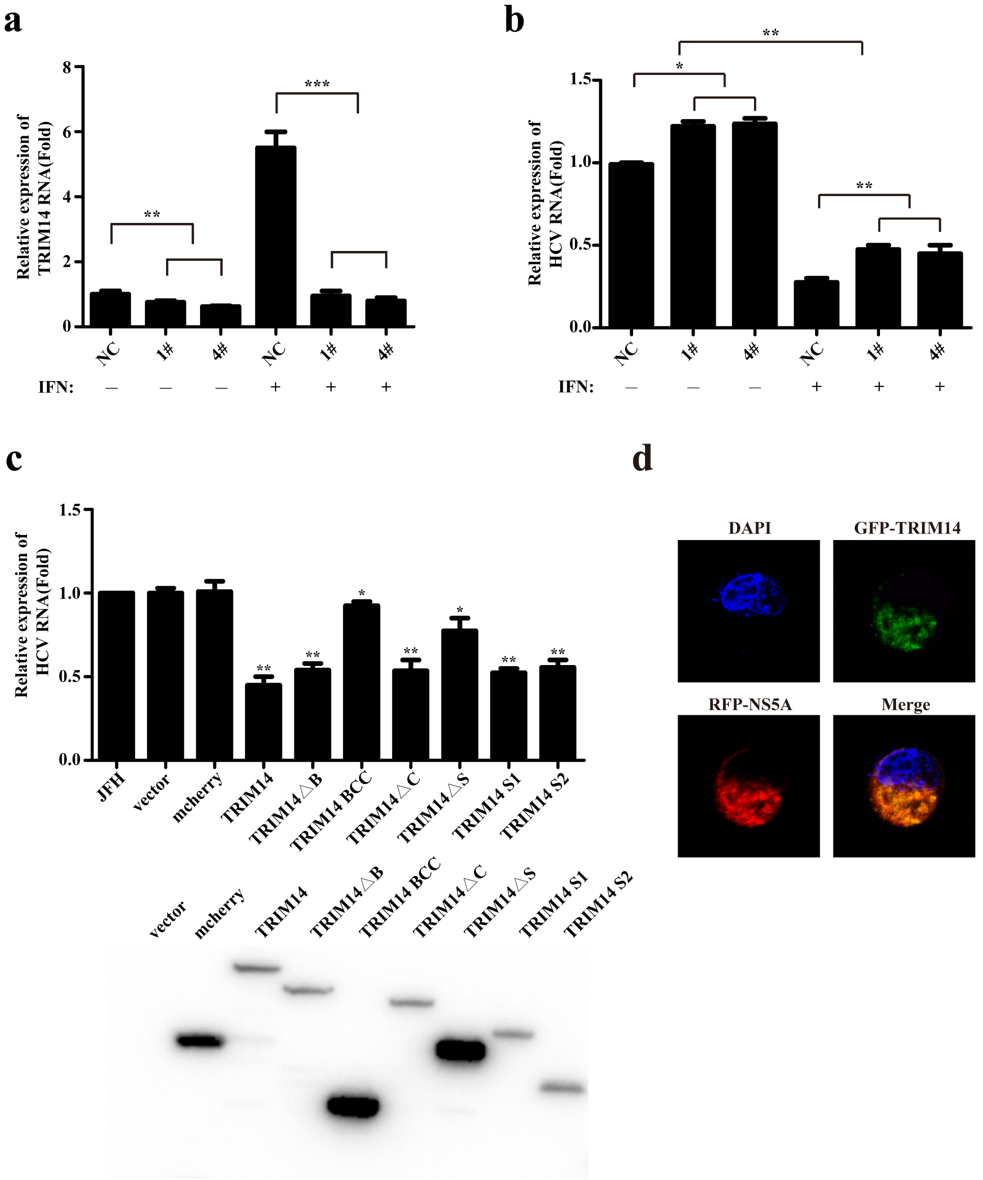

Supplemental Figure2. TRIM14 inhibits HCV replication

(a)-(b) JFH cells were transfected with indicated siRNA oligos 1# or 4# were treated with or without IFN for 24 hours and analyzed by quantitative real-time PCR to determine the relative expression of TRIM14 and HCV RNA. **(c)** Effect of TRIM14 truncations on HCV replication. The TRIM14 truncations expression plasmids or a control plasmid were transfected into TRIM14 knockout JFH cells. After 48 hours, total RNA was analyzed for expression of HCV replicon and quantified by real-time PCR. Immunoblot analysis extracts of cells which were transfected with plasmids of pFlag-TRIM14 truncations (TRIM14ΔB, TRIM14BCC, TRIM14ΔC, TRIM14ΔS, TRIM14S1, TRIM14S2). **(d)** Colocalization of TRIM14 with NS5A. Huh7 cells were co-transfected with GFP-TRIM14 and RFP-NS5A expression plasmids for 24 hours, then cell nucleus were stained with DAPI, then cells were detected by laser confocal microscopy. Red fluOrescence shows NS5A staining, while green fluorescence detects TRIM14.

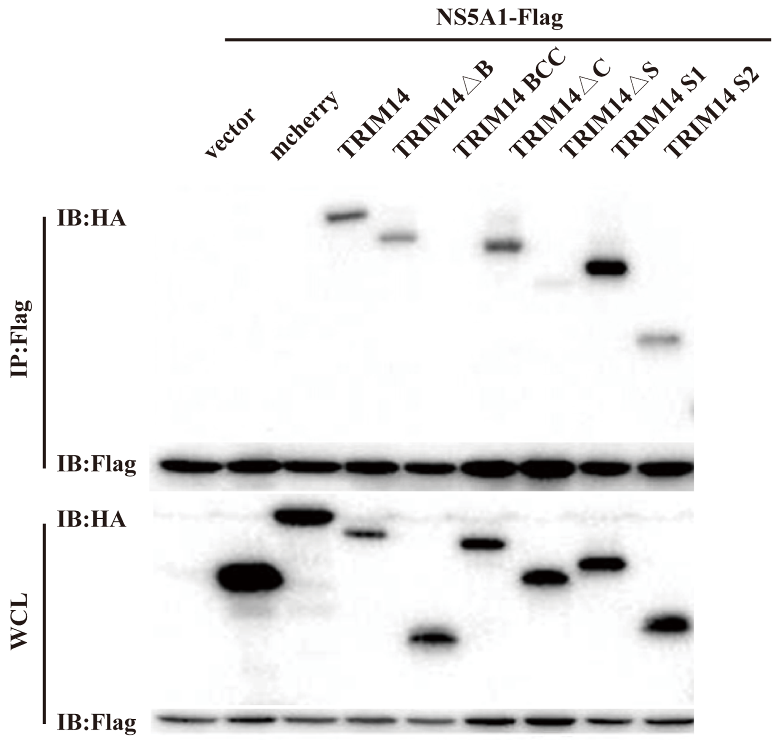

Supplemental Figure 3. TRIM14 and TRIM14 truncations interact with NS5A1

Immunoblot analysis of extracts from HEK293T cells which were transfected with the plasmids of pFlag-NS5A1 and pHA-TRIM14 truncations (TRIM14ΔB, TRIM14BCC, TRIM14ΔC, TRIM14ΔS, TRIM14S1, TRIM14S2), mcherry as a negative control. Co-IP assays were performed using cell lysates from cells expressing the indicated constructs.

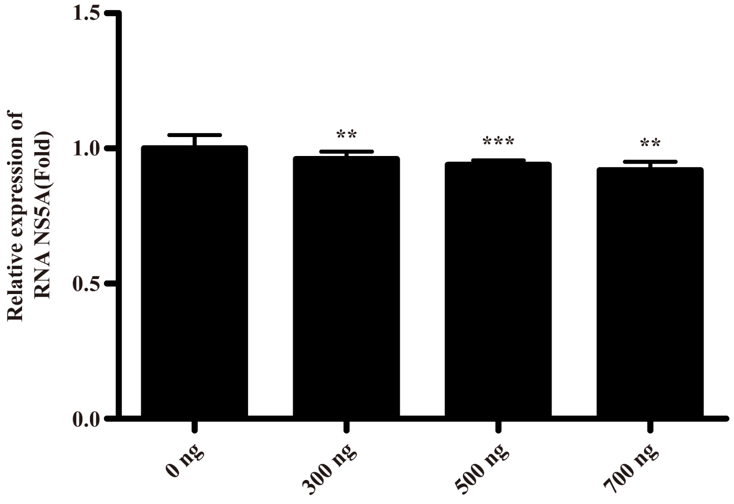

Supplemental Figure 4. TRIM14 does not affect NS5A mRNA stability.

HEK293T cells were transfected with pFlag-NS5A1 plasmids and an increasing amount (0ng, 300ng, 500ng or 700ng, wedges) of expression plasmids for pFlag-TRIM14. After 24 hours, cells were collected and total RNA was analyzed for expres- sion of NS5A1 and quantified by real-time PCR.

a

JFH cell

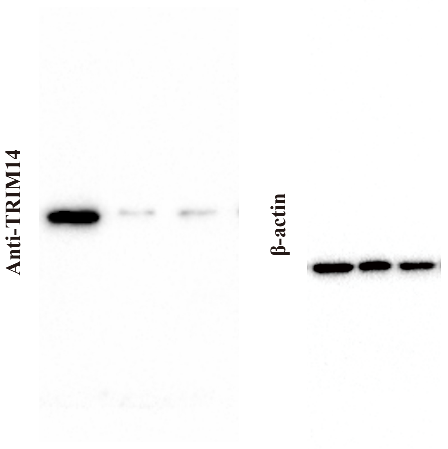

From Figure 1b

b

Huh7 cell

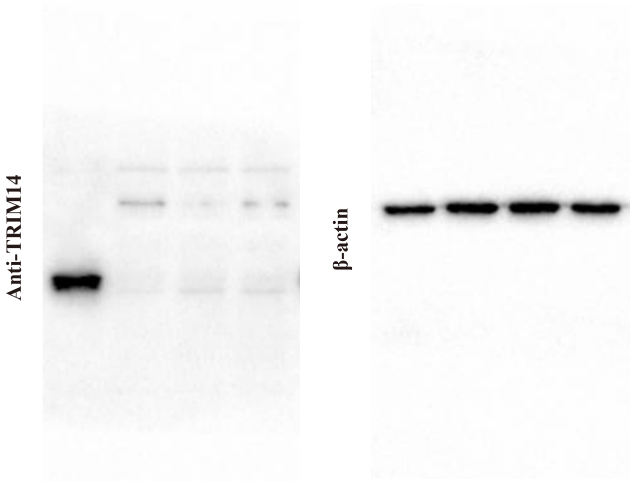

From Figure 1c

c

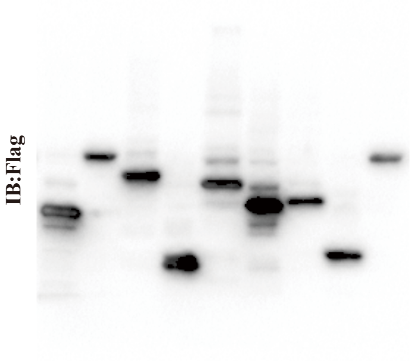

From Figure 2d

d

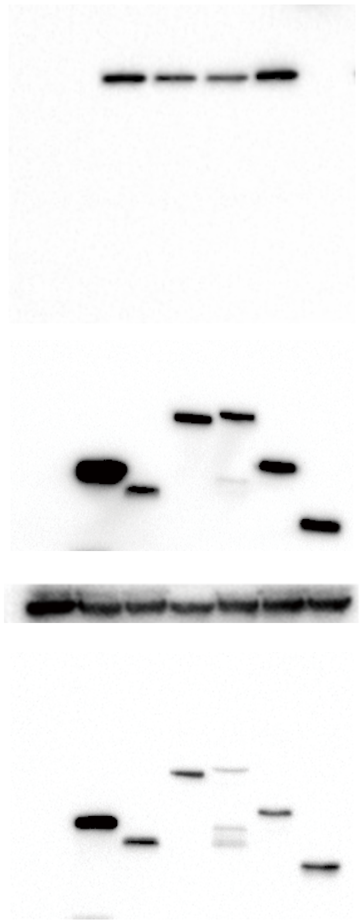

From Figure 3d

e

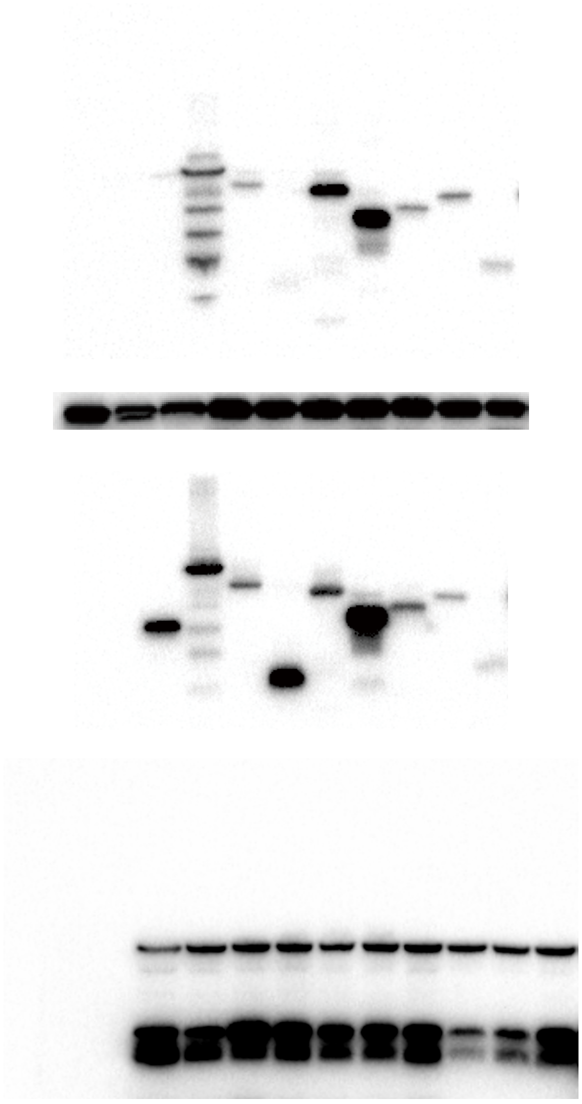

From Figure 3f

a

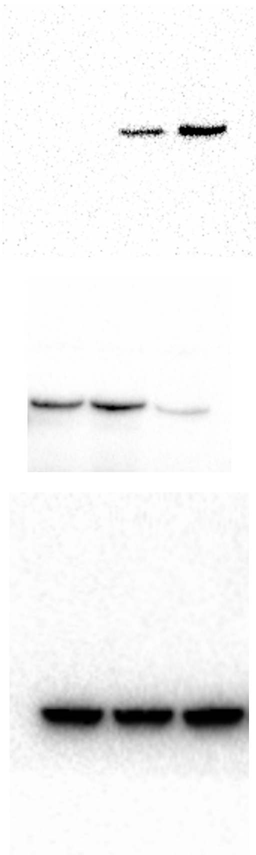

From Figure 4a

b

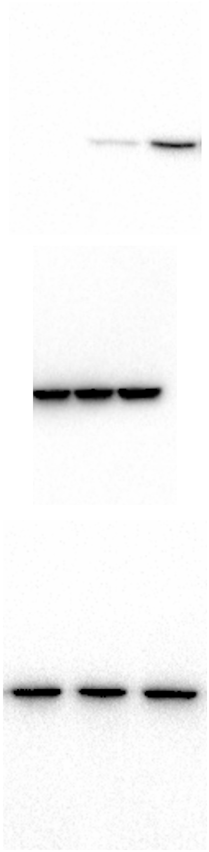

From Figure 4b

c

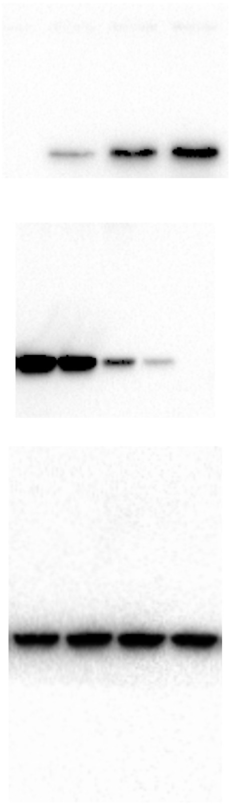

From Figure 4c

d

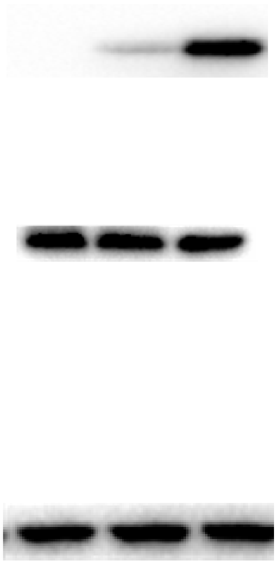

From Figure 4d

e

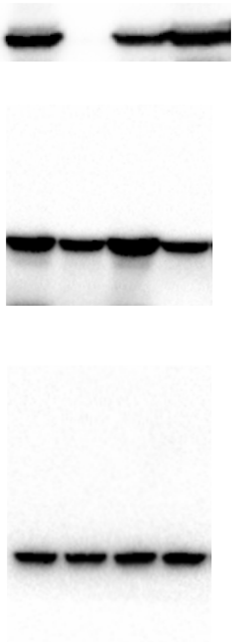

From Figure 4e

f

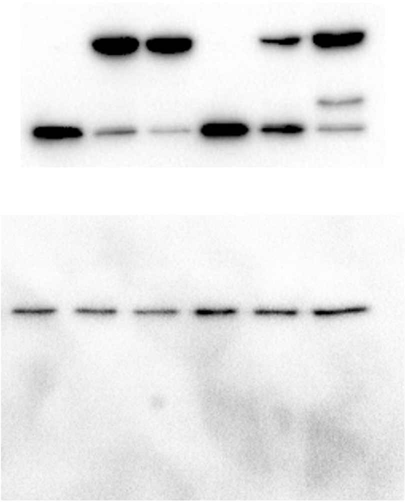

From Figure 4f

a

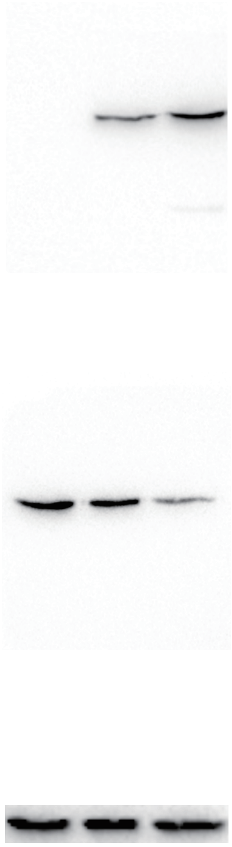

From Figure 5a

b

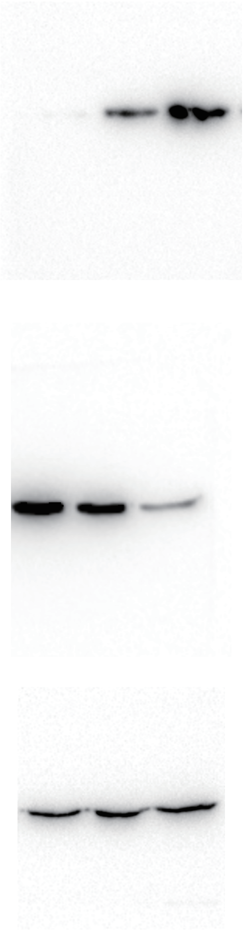

From Figure 5b

c

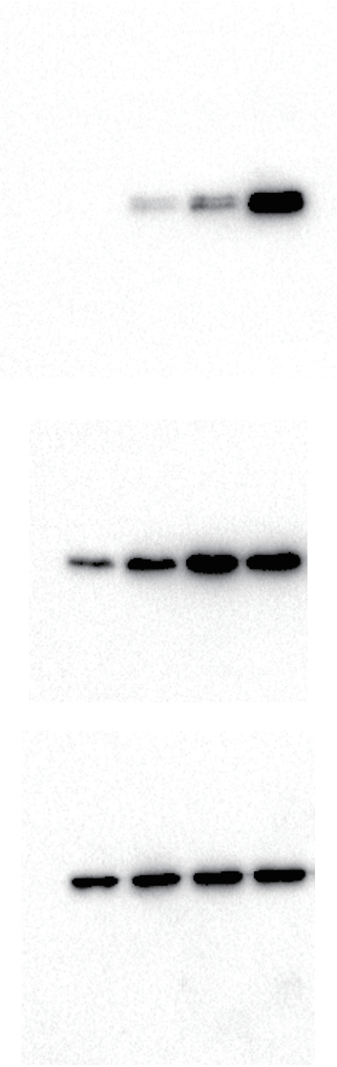

From Figure 5c

d

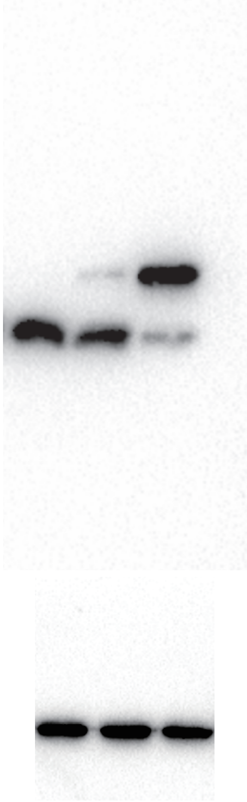

From Figure 5d

e

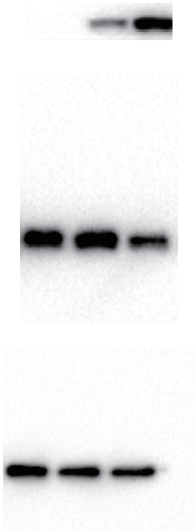

From Figure 5e

f

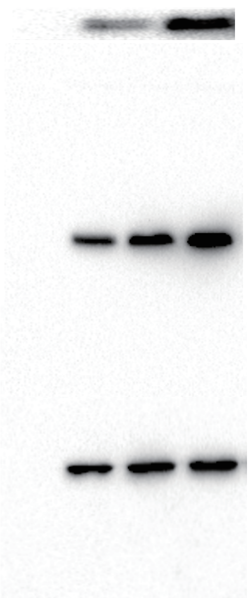

From Figure 5f

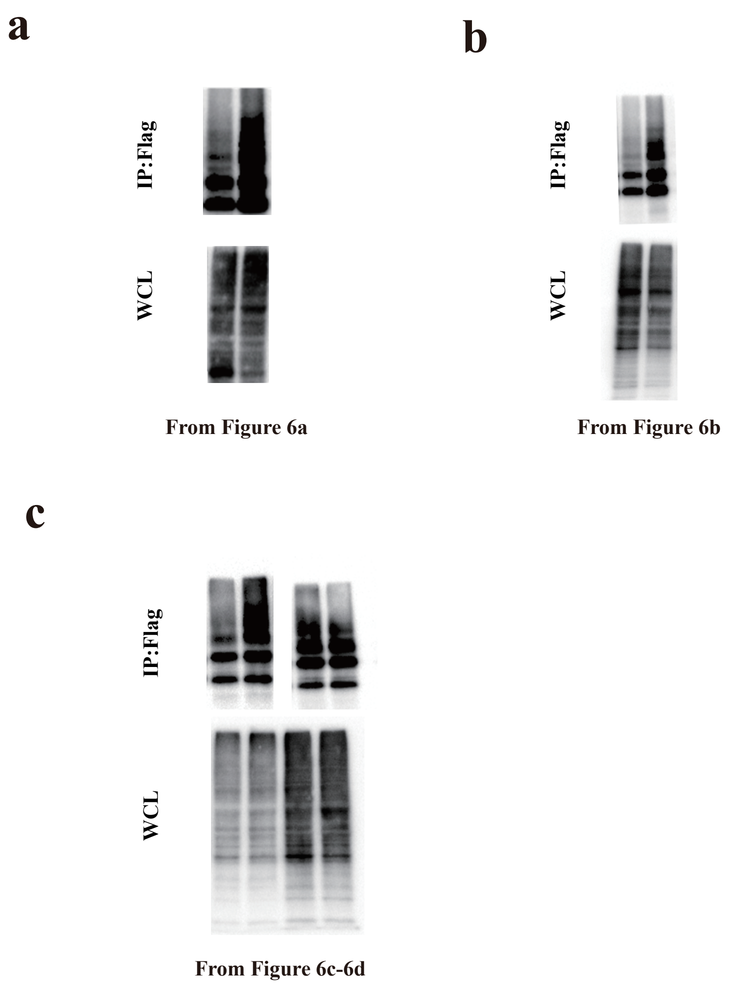

Supplemental Figure 5-8. Original panel for each western blot

In some western blot experiments, one PVDF membrane was need to incubation 3 or 4 kinds of antibodies, so according to the size of the protein, the PVDF membrane was cut into small strips in experiments, then they were incubated with corresponding antibody and collected exposure results.
